# High Intensity Interval- vs Moderate Intensity- Training for Improving Cardiometabolic Health in Overweight or Obese Males: A Randomized Controlled Trial

**DOI:** 10.1371/journal.pone.0138853

**Published:** 2015-10-21

**Authors:** Gordon Fisher, Andrew W. Brown, Michelle M. Bohan Brown, Amy Alcorn, Corey Noles, Leah Winwood, Holly Resuehr, Brandon George, Madeline M. Jeansonne, David B. Allison

**Affiliations:** 1 Department of Human Studies, University of Alabama at Birmingham, Birmingham, AL, United States of America; 2 Nutrition and Obesity Research Center, University of Alabama at Birmingham, Birmingham, AL, United States of America; 3 Office of Energetics, University of Alabama at Birmingham, Birmingham, AL, United States of America; 4 Department of Nutrition Sciences, University of Alabama at Birmingham, Birmingham, AL, United States of America; 5 Department of Food, Nutrition, and Packaging Sciences, Clemson University, Clemson, SC, United States of America; Texas A&M University, UNITED STATES

## Abstract

**Purpose:**

To compare the effects of six weeks of high intensity interval training (HIIT) vs continuous moderate intensity training (MIT) for improving body composition, insulin sensitivity (S_I_), blood pressure, blood lipids, and cardiovascular fitness in a cohort of sedentary overweight or obese young men. We hypothesized that HIIT would result in similar improvements in body composition, cardiovascular fitness, blood lipids, and S_I_ as compared to the MIT group, despite requiring only one hour of activity per week compared to five hours per week for the MIT group.

**Methods:**

28 sedentary overweight or obese men (age, 20 ± 1.5 years, body mass index 29.5 ± 3.3 kg/m^2^) participated in a six week exercise treatment. Participants were randomly assigned to HIIT or MIT and evaluated at baseline and post-training. DXA was used to assess body composition, graded treadmill exercise test to measure cardiovascular fitness, oral glucose tolerance to measure S_I_, nuclear magnetic resonance spectroscopy to assess lipoprotein particles, and automatic auscultation to measure blood pressure.

**Results:**

A greater improvement in VO_2peak_ was observed in MIT compared to HIIT (11.1% vs 2.83%, P = 0.0185) in the complete-case analysis. No differences were seen in the intention to treat analysis, and no other group differences were observed. Both exercise conditions were associated with temporal improvements in % body fat, total cholesterol, medium VLDL, medium HDL, triglycerides, S_I_, and VO_2peak_ (P < 0.05).

**Conclusion:**

Participation in HIIT or MIT exercise training displayed: 1) improved S_I_, 2) reduced blood lipids, 3) decreased % body fat, and 4) improved cardiovascular fitness. While both exercise groups led to similar improvements for most cardiometabolic risk factors assessed, MIT led to a greater improvement in overall cardiovascular fitness. Overall, these observations suggest that a relatively short duration of either HIIT or MIT training may improve cardiometabolic risk factors in previously sedentary overweight or obese young men, with no clear advantage between these two specific regimes (Clinical Trial Registry number NCT01935323).

**Trial Registration:**

ClinicalTrials.gov NCT01935323

## Introduction

The prevalence of obesity has increased worldwide among both children and adults, and obesity is associated with an increased risk for cardiovascular diseases (CVDs) [1, 2]. Longitudinal data have shown that adolescent adiposity and fat distribution tracks into adulthood, as 50–80% of obese adults have been shown to be also obese as adolescents [3, 4]. CVDs are associated with alterations in blood lipids, such as elevated triglycerides (TG), low high density lipoprotein cholesterol (HDL-C), elevated low density lipoprotein cholesterol (LDL-C), and alterations in lipoprotein subclasses [5, 6]. In addition to dyslipidemia; hypertension, low cardiovascular fitness, obesity, and type 2 diabetes are also known modifiable risk factors that are associated with risk for CVDs [2, 7]. Furthermore, it has becoming increasingly clear that low cardiovascular fitness may exacerbate CVD mortality risk, and that increasing peak cardiovascular fitness to > 5 peak metabolic equivalents (MET) can reduce and perhaps eliminate mortality rate associated with dyslipidemia, obesity, TDM, and hypertension [8]. Thus, it is important to better understand how exercise interventions affect risk factors associated with cardiometabolic diseases, especially in the adolescent and young adult population.

Despite the well-established benefits of routine physical activity for improving cardiometabolic health, it remains difficult for health professionals to get individuals to adhere to current physical activity guidelines of at least 30 min per day of moderate intensity exercise 5 days per week or vigorous exercise for 20 min per day 3 days a week [9]. Therefore, given that ‘lack of time’ is the most commonly cited barrier to exercise adherence, more recent studies have focused on identifying a more time-efficient mode of exercise training. Low-volume, high-intensity interval training (HIIT) (consisting of 30 second all out sprints separated by recovery intervals) is a mode of training that requires very little time, yet in many studies shows similar improvements in reducing cardiometabolic risk factors as traditional moderate-intensity continuous training (MIT) programs despite only requiring 10–20% of the time commitment [10–12]. Given these findings, HIIT may be an ideal mode of training in adolescents and young adults who often struggle to find the time to exercise as they transition from participation in youth and team sports and embark on their college education or become working professionals.

A growing body of evidence has demonstrated comparable or superior improvements in cardiometabolic health outcomes using HIIT as compared to MIT [12]. For example, HIIT interventions have shown similar improvements in skeletal muscle metabolic adaptations, cardiovascular fitness, vascular function, and body composition as compared to a much higher volume of MIT [10, 11]. Furthermore, Trapp et al. compared 15 weeks of HIIT versus MIT and found a significant decrease in subcutaneous fat and increase in fat-free mass in the HIIT group whereas no change in subcutaneous fat or fat-free mass occurred in the MIT group [13]. Additionally, several recent studies that did not include a MIT comparison group have demonstrated the ability of HIIT to reduce whole body and abdominal fat mass while increasing fat-free mass [14, 15]. Thus, based on these observations HIIT may provide a more optimal exercise stimulus to promote fat loss while preserving fat free mass during weight loss.

To date, there are limited data on the benefits of HIIT on glycemic control. A few studies have shown improvements in insulin sensitivity, fasting glucose, fasting insulin, or 24-hr glucose concentrations using continuous glucose monitoring (CGM) [16–18], however only two of these studies included a direct comparison with a MIT exercise group [17, 18]. Of these two studies, one found improvements in both the HIIT and MIT groups over time [17], while the other only found an improvement in the HIIT group over time with no change in the MIT group [18]. Additionally, several studies that did not include a MIT group using sprint interval training and other modes of HIIT have demonstrated an improvement in post-prandial glucose metabolism and glucose tolerance acutely, however the chronic effects of this mode of exercise on glucose regulation is less understood [16, 19]. Thus, these data demonstrate the benefits of HIIT but the relative health benefits between HIIT and MIT are less clear. If comparable, HIIT may be a ‘time efficient’ mode of exercise training that may be ideal for adolescents or young adults who struggle with time management or other obstacles to adhering to current exercise recommendations.

Thus, the purpose of this study was to compare the effects of six weeks of HIIT vs MIT for improving body composition, insulin sensitivity, blood pressure, blood lipids, and cardiovascular fitness in a cohort of sedentary overweight young men (age 17–22 years old). We hypothesized that HIIT would result in similar improvements in body composition, cardiovascular fitness, blood lipids, and insulin sensitivity as compared to the MIT group, despite requiring only one hour of activity per week compared to five hours per week for the MIT group.

## Methods

### Participants

Participants were 28 sedentary overweight or obese (BMI 25–35 kg/m^2^) men between the ages of 17–22 years. Inclusion criteria were BMI 25–35 kg/m^2^, sedentary (< 30 minutes of structured activity per week), normal glucose tolerance (fasting glucose < 100 mg /dL), and no use of medications known to affect body composition or metabolism. All participants were nonsmokers. The study was approved by the Institutional Review Board for human use at the University of Alabama at Birmingham (UAB) **(S1 Protocol)**. All participants provided written informed consent before participating in the study. Parents or legal guardians provided written consent for participants between age 17–18 years old. ClinicalTrials.gov registration was done before participant enrollment, but due to an undetected submission error finalization was not confirmed until 11 participants were enrolled. IRB for this study was approved 10 JAN 2013. The first enrolled participant was screened on 22 FEB 2013, and the last participant completed on 3 NOV 2014. All other relevant trials conducted by the investigators have been successfully registered.

### Study design

**Fig 1:**
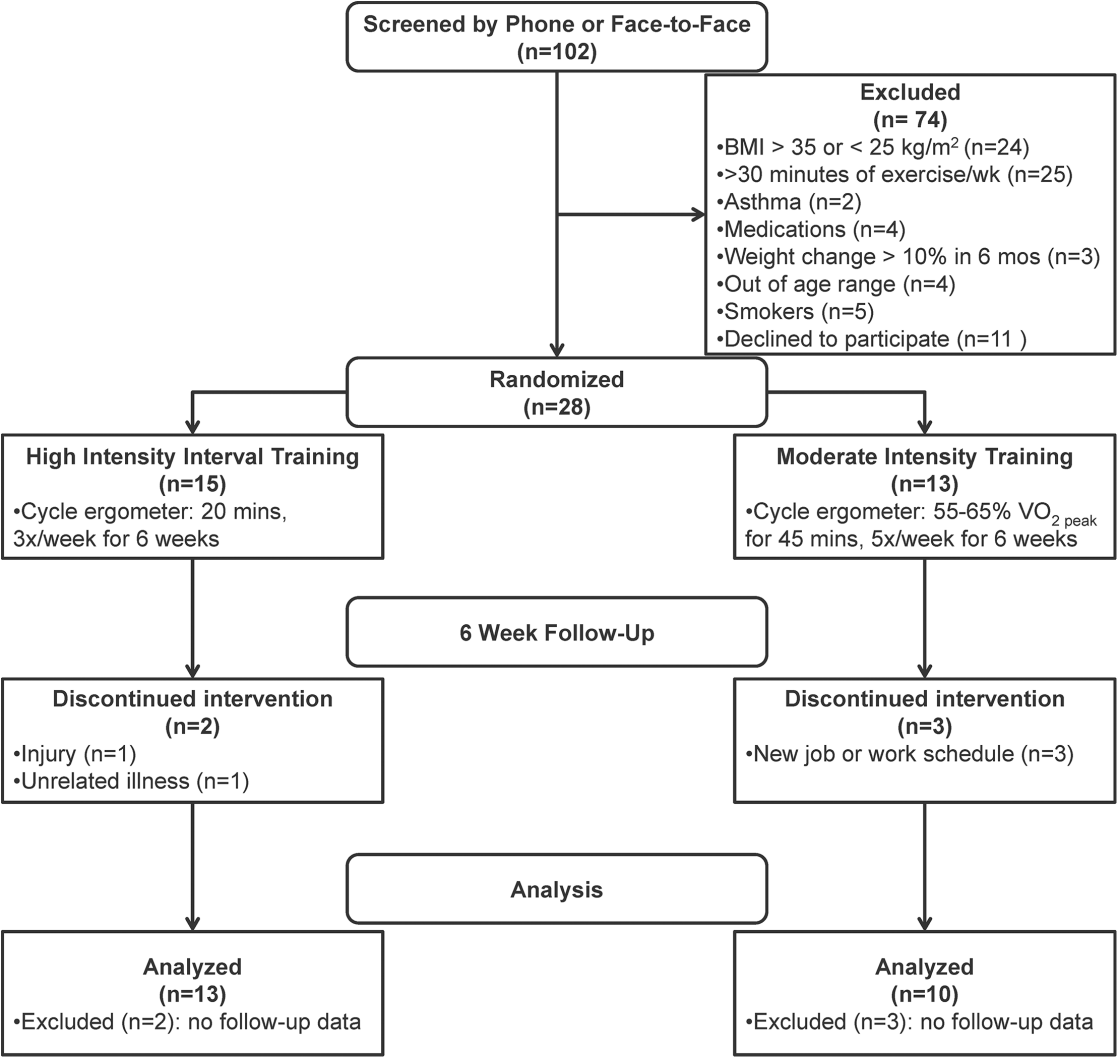
Consort Flow Diagram of Study.

This was a six-week, single site, two parallel arm, randomized, controlled trial comparing the effectiveness of a HIIT versus a MIT program on cardiovascular and metabolic health outcomes in overweight or obese adolescent men registered with ClinicalTrials.gov (registry number NCT01935323). Participants were randomly assigned to either HIIT or MIT training groups. Random treatment assignment was conducted using pseudo-random sampling in R by a statistician that had no contact with the participants, with an arbitrary but fixed seed set to 1234. Random assignments were created in 3 waves of 12, with balanced randomization within each wave. Assignments were placed in opaque, numbered envelopes and distributed to participants sequentially after a participant passed the screening tests, thereby enforcing allocation concealment from both the participant and investigators until the envelope was opened. 28 participants enrolled in the study (n = 13 for MIT and n = 15 for HIIT). In total, five participants dropped out of the study during the intervention, three from the MIT group and two from the HIIT group (**CONSORT Flow Diagram, Fig 1**). Participants underwent baseline assessment, were assigned to six weeks of HIIT or MIT exercise training, and were assessed again at post-exercise training.

### Pre-training testing protocol

Eligible participants attended four baseline visits. Day 1) following an overnight fast, resting metabolic rate, body composition, and blood pressure were assessed; Day 2) an oral glucose tolerance test (OGTT) was conducted, and baseline blood samples were used for lipoprotein analysis; Day 3) participants performed an incremental peak VO_2_ test on an electronically-braked cycle ergometer (Monark 894 E). Each participant pedaled at 50 watts for 3 minutes, workload was then increased 25 watts per minute until volitional exhaustion. Oxygen uptake and was measured continuously by open-circuit spirometry and analyzed using a Sensormedics metabolic testing system (model 2900, Yorba Linda, CA, USA). The highest VO_2_ achieved during the last stage was recorded as the VO_2 peak_, Day 4): participants performed a 30-s Wingate maximal anaerobic power test on a cycle ergometer with resistance determined by body weight (0.075 kg/kg body wt), with data collected and analyzed electronically using the Monark Anaerobic Test Software. VO_2 peak_ and Wingate assessments were separated by at least 24 hrs.

### Exercise training

HIIT was performed on an electronically-braked cycle ergometer (Quinton Excalibur, Quinton Instrument Company, Bothell, WA). Participants performed a 20-minute protocol, consisting of four minutes of cycling at 15% of maximum anaerobic power (Max-AP) (defined as the peak power achieved during the Wingate Test) followed by 30 seconds at 85% of Max-AP. These workloads were based upon pre-trial Wingate tests. This cycle was repeated four times within each protocol, ending with two minutes at 15% of Max-AP. This was performed 3d/wk for 6 wks, with at least 24 hrs between each session. MIT performed 45–60 min of continuous cycling at 55–65% of VO_2peak_ (graduated over time to 60 min and 65% as participants were able) on a Monark cycle ergometer. Workload was based upon pre-trial VO_2peak_ testing. MIT exercise was performed 5d/wk for 6 wks. Heart rate was monitored for each exercise session. Heart rate was recorded every 5 minutes during MIT and during minute 4 of recovery and immediately following completion of the 30 second work interval for HIIT. Exercise training data for each group are shown in (**Table 1**).

**Table 1:** Exercise Training Data and Weekly Time Commitment.

Variable	HIIT Group	MIT Group
**Protocol**	30s x 4 Repeats; 4 min Rest	45–60 min Cycling
**Frequency**	3 Sessions per Week	5 Sessions per Week
**Workload (watts)**	Interval: 85% Peak Power, 810 ± 250 W	55–65% VO_2peak_: 138 ± 13 W
	Recovery: 15% Peak Power, 140± 20 W	
**HR (bpm)**	Interval: 178 ± 9 bpm	158 ± 11 bpm
	Recovery: 140 ± 13 bpm	
**Weekly Training Time**	Interval: 6 min	3.75–5.00 hours
	Recovery: 54 min	
	Total: 60 min	

Values are means ± SD; MIT *n* = 10 and HIIT *n* = 13 subjects.

### Body composition

Total body composition, including total fat mass, percent body fat, and fat-free mass were measured by dual-energy X-ray absorptiometry (Lunar Prodigy; GE Healthcare Lunar, Madison, WI). Subjects were scanned in light clothing while lying flat on their backs with arms at their sides. The scans were analyzed with the use of ADULT software version 1.33 (Lunar Radiation).

### Resting Blood Pressure

Resting blood pressure (systolic and diastolic) was taken by automatic auscultation (Omron Blood Pressure Monitor, model HEM-780; Omron Healthcare, Inc 1200 Lakeside Dr. Bannockburn, IL) while lying in a supine position. Readings were taken after 12 hours of fasting between 7:00–9:00 AM.

### Oral glucose tolerance test and insulin sensitivity

Whole body insulin sensitivity (S_I_) was assessed using an oral glucose tolerance test (OGTT) on an in-patient basis in the Clinical Research Unit of UAB’s Center for Clinical and Translational Sciences (CCTS) after an overnight fast. The final OGTT was performed at least 24 hrs following the last exercise session for each group. Prior to testing, a flexible intravenous catheter was placed in the antecubital space of the arm. Two baseline blood samples were taken over a 20-min period to determine basal glucose and insulin. At time 0 each subject consumed a 75-g oral glucose load within 5 min. Blood samples were collected at 10, 20, 30, 60, 90, and 120 min after glucose ingestion for measurement of plasma glucose and plasma insulin. Blood was immediately centrifuged, separated for serum, and frozen at −80°C until analysis. Assays were performed in the CCTS Metabolism Core. Serum glucose assays were performed on an automated glucose analyzer (Sirrus analyzer; Stanbio Laboratory, Boerne, TX), and serum insulin was measured using an immunofluorescent method with an AIA-600 II analyzer (TOSOH Bioscience, South San Francisco, CA) per manufacturers' instructions. Whole body insulin sensitivity (S_I_) was calculated from OGTT data using the Matsuda index [20]. A second measure of insulin sensitivity was calculated using the Quantitative Insulin Sensitivity Check Index (QUICKI) [21]. QUICKI was calculated as 1/[log glucose (mg/dL) + log insulin (μU/ml)].

Insulin resistance (IR) was assessed using the Homeostasis Model of Assessment of Insulin Resistance (HOMA-IR). HOMA-IR was calculated as [fasting insulin (μU/ml) x fasting glucose (mmol/L)]/22.5.

### Resting energy expenditure

Resting energy expenditure (REE) was determined between 7:00 and 9:00 a.m. Subjects remained awake in a quiet, softly lit, well ventilated room in which temperature was maintained between 22 and 24°C. Subjects lay supine on a comfortable bed and oxygen uptake was measured using a ventilated hood system. After resting for 15 minutes, oxygen uptake was measured for 30 minutes with a computerized, open-circuit indirect calorimetry system (Delta Trac II, Sensor Medics, Yorba, CA, USA). The last 20 minutes of steady state data were used for analysis.

### Lipoprotein Analysis

Fasting blood samples were collected via antecubital venipuncture into K_2_EDTA-containing vacutainers. Tubes were kept on ice (no more than 20 minutes) until centrifuged at 3,000 x g for 20 min at 4°C. A volume of 0.5 mL of plasma was stored in 1.5 mL microcentrifuge tubes at -80°C until shipped to LipoScience for nuclear magnetic resonance spectroscopy (NMR) for lipoprotein profile analysis. Samples were shipped per LipoScience instructions (overnight on dry ice) and analyzed via LipoScience’s proton NMR lipoprotein analysis methodology based on deconvoluting methyl proton signals centered at 0.8 ppm [22]. NMR spectrum analysis provides estimates of lipoprotein particle size (nm) and concentration (nM or μM). Specifically: small, medium, and large HDL; small and large LDL; IDL; small and medium VLDL; and combined large VLDL and chylomicron particles. Because participants were fasting, the contribution of chylomicrons to this latter category should be minimal.

### Additional Blood Analyses

Laboratory analyses were conducted in the Core Laboratory of the CRU, Nutrition Obesity Research Center, and Diabetes Research Center. Total cholesterol, HDL-Cl, and triglycerides were measured using a SIRRUS analyzer (Stanbio Laboratory, Boerne, TX); LDL-C was calculated using the method of Friedewald [23].

### Post-training testing protocol

Post-training assessments for metabolic rate, body composition, blood pressure, and OGTT were conducted at least 24-hrs after the final training session. Post-training VO_2peak_ and Wingate assessments were completed within 72-hrs following the final training session.

### Statistical Analysis

All calculations were done using SAS 9.4 (SAS Institute Inc., Cary, NC). All data are presented as mean ± standard deviation. Differences between groups at baseline were assessed using either a two-sample t-test for continuous variables or a chi-square test for the categorical variable race. As a validity check to confirm expected metabolic improvements of being in an exercise study with two active treatments, the overall pre-post differences were tested using a paired t-test. The primary analysis assessed the treatment effect on the change over time using an ANCOVA model with the baseline outcome value and proportion of scheduled workouts completed (18 for HIIT, 30 for MIT) as covariates. Subjects with missing values for follow-up were excluded from the primary analysis (i.e., a complete-case analysis), as this study was designed as a small efficacy study and the effect of either treatment arm is dependent on active participation in the study. As a sensitivity analysis, an intention to treat analysis (ITT) was conducted in which the data were analyzed as randomized using the full information restricted maximum likelihood in a repeated measures model with Kenward-Roger correction for degrees of freedom when testing the treatment-by-time interaction. The validity check for expected pre-post differences was verified by examination of the main effect of time in this model. No clear violations of the normality assumption were observed. No corrections were made for multiple comparisons.

## Results

Twenty-eight participants enrolled in the study. The mean age of the participants at baseline was 20 ± 1.5 yrs; mean BMI 29.5 ± 3.3 kg/m^2^. The ethnic composition was 18 non-Hispanic Caucasian, 8 African-American, and 2 Hispanic. Of these, five participants dropped out of the study during the intervention, three from the MIT group and two from the HIIT group. Reasons for discontinuing the study included transportation problems, relocation, an illness, and an injury unrelated to the study treatment.

Exercise training data and weekly time commitment by exercise group are shown in **Table 1**. Average peak power was 810 ± 249 watts during intervals and 140 ± 21 watts during recovery for HIIT. Average peak power for the MIT was 138 ± 13 watts. These workloads corresponded to 325% and 56% of VO_2peak_ during the HIIT sessions, and 55% of VO_2peak_ during MIT sessions. Average heart rate during HIIT was 178 ± 21 BPM for intervals and 153 ± 14 BPM during recovery, and 159 ± 12 BPM during MIT.

Baseline characteristics by training group are shown in **Table 2**. Although participants were randomized to each exercise group, HIIT participants had a significantly greater percent body fat compared to the MIT group at baseline (P = 0.0454; 1 of 23 baseline tests). No other statistically significant baseline differences were observed.

**Table 2:** Baseline characteristics between HIIT and MIT.

Variable	MIT (N = 13)	HIIT (N = 15)	p-Value
Age (yrs)	20.0 (1.5)	20.0 (1.5)	0.7321
African-American	6 (46%)	2 (13%)	0.0957
Weight (kg)	89.7 (15.8)	94.3 (12.1)	0.4049
BMI (kg/m^2^)	29.0 (3.4)	30.0 (3.1)	0.4215
Percent fat (%)	29.49 (5.08)	34.08 (6.45)	**0.0454**
VO_2_ Peak (ml/kg/min)	34.95 (6.46)	35.72 (6.22)	0.7505
Peak Power (watts)	1022.4 (179.8)	891.0 (296.6)	0.1638
Resting Metabolic Rate (kcal/day)	1806.3 (215.3)	1835.4 (232.5)	0.7339
Total cholesterol (mg/dL)	163.0 (26.6)	169.3 (25.4)	0.5268
Triglycerides (mg/dL)	128.3 (103.7)	122.5 (55.9)	0.8228
HDL-cholesterol (mg/dL)	50.1 (9.2)	46.9 (10.6)	0.3985
LDL-cholesterol (mg/dL)	90.6 (16.8)	100.1 (19.4)	0.1762
Large VLDL Particles	5.18 (8.6)	3.3 (2.9)	0.5157
Medium VLDL Particles	25.81 (25.5)	24.55 (18.3)	0.8068
Small VLDL Particles	23.53 (11.5)	25.73 (16.3)	0.2355
Large HDL Particles	5.14 (2.7)	4.11 (2.2)	0.1665
Medium HDL Particles	11.63 (5.3)	13.75 (3.6)	0.6737
Small HDL Particles	15.53 (4.9)	12.91 (3.4)	0.355
SBP (mm Hg)	126.5 (12.7)	129.5 (9.7)	0.4890
DBP (mm Hg)	71.2 (6.9)	68.8 (7.0)	0.3727
Insulin Sensitivity (S_I_)	4.57 (3.20)	3.60 (1.89)	0.3504
HOMA-IR	2.73 (1.55)	2.73 (1.33)	0.9990
QUICKI	0.3397 (0.0349)	0.3363 (0.0271)	0.7814

Table 2: Baseline characteristics of the two treatment groups. Values are Mean (SD) or N (%). Continuous variables were compared with a two-sample t-test with a Satterthwaite adjustment. Categorical variables were compared using Fisher’s exact test. Boldface values indicate significance differences (P < 0.05).

Because there were two active treatment groups in this study, it was prudent to assess if there was an overall benefit metabolic outcomes over time. The results of this descriptive (i.e. not inferential) analysis are given in **Table 3**. Randomization to either exercise condition was associated with improvements in % fat (p = 0.0087), total cholesterol (p = 0.0236), medium VLDL (p = 0.0323), medium HDL (p = 0.0157), triglycerides (p = 0.0129), S_I_ (p = 0.0374), QUICKI (p = 0.0254), and VO_2peak_ (p = 0.0164). The results of the ITT analysis for the overall change over time were mostly similar to the primary complete case analysis, although QUICKI became non-significant.

**Table 3:** Results for body composition and physiological variables over time.

	Change Score (Mean (SD))			Test of Change Over Time		Test of Treatment Effects	
	MIT	HIIT	Overall	Completers^1^	ITT^2^	Completers^1^	ITT^2^
Weight (kg)	-1.09 (2.42)	-0.82 (2.57)	-0.94 (2.45)	0.0794	0.0792	0.3686	0.7351
BMI (kg/m^2^)	-0.35 (0.75)	-0.26 (0.83)	-0.29 (0.78)	0.0828	0.0832	0.4792	0.7233
Percent fat (%)	-1.28 (2.20)	-0.88 (1.41)	-1.06 (1.76)	**0.0087**	**0.0083**	0.8996	0.6005
VO_2_ Peak (ml/kg/min)	3.40 (3.36)	0.84 (3.50)	1.95 (3.60)	**0.0164**	**0.0147**	**0.0185**	0.1032
Peak Power (watts)	-20.6 (87.9)	38.6 (106.2)	14.4 (101.4)	0.513	0.5353	0.1865	0.1065
Resting Metabolic Rate (kcal/d)	25.0 (173.3)	-13.0 (113.3)	3.5 (140.2)	0.9052	0.8822	0.8972	0.8026
Total cholesterol (mg/dL)	-12.9 (26.4)	-9.2 (17.6)	-10.8 (21.4)	**0.0236**	**0.0206**	0.7129	0.6488
Triglycerides (mg/dL)	-15.7 (47.4)	-15.3 (46.3)	-26.4 (46.8)	**0.0129**	**0.0126**	0.5875	0.5273
HDL-cholesterol (mg/dL)	-2.0 (5.1)	-1.4 (7.9)	-1.65 (6.71)	0.2504	0.2451	0.1997	0.9237
LDL-cholesterol (mg/dL)	-7.8 (18.6)	-4.78 (16.9)	-6.08 (17.31)	0.1063	0.0941	0.3612	0.4946
Large VLDL Particles	-0.31 (2.6)	-1.28 (2.9)	-0.9 (2.8)	0.1544	0.1551	0.4970	0.3977
Medium VLDL Particles	-5.69 (19.9)	-8.41 (10.9)	-7.2 (15.2)	**0.0323**	**0.0318**	0.9738	0.7571
Small VLDL Particles	-2.42 (15.3)	5.48 14.5)	2.0 (15.0)	0.5204	0.5162	0.3064	0.2455
Large HDL Particles	0.15 (1.25)	0.10 (1.1)	0.1 (1.2)	0.6169	0.6255	0.1112	0.7999
Medium HDL Particles	-1.79 (5.3)	-3.35 (3.1)	-2.7 (4.9)	**0.0157**	**0.0164**	0.4912	0.3205
Small HDL Particles	0.58 (5.56)	2.54 (3.7)	1.7 (4.6)	0.0923	0.1049	0.3771	0.1750
SBP (mm Hg)	-4.6 (11.4)	-0.3 (11.1)	-2.2 (11.2)	0.3614	0.3682	0.6430	0.2506
DBP (mm Hg)	-4.6 (6.4)	1.6 (7.7)	-1.1 (7.7)	0.5049	0.4824	0.2495	**0.0361**
Insulin Sensitivity (S_I_)	0.183 (0.799)	1.154 (1.826)	0.71 (1.50)	**0.0374**	**0.0401**	0.2754	0.1199
HOMA-IR	-0.27 (0.40)	0.16 (2.35)	-0.03 (1.73)	0.9264	0.9434	0.4627	0.6638
QUICKI	0.0059 (0.0099)	0.0043 (0.0340)	0.0050 (0.0254)	**0.0254**	0.4316	0.9184	0.9564

Table 3: Changes in variables (Final–Baseline) for the MIT and HIIT groups over time. Change scores are Mean (SD). Reported p-values are for tests of an overall change over time among all subjects, and for tests of a treatment effect between MIT and HIIT groups over time.

*Boldface values indicate significance differences (P < 0.05).

^1^Only participants with complete data are included in the complete case analysis.

^2^Intent to treat (ITT) analysis: missing follow-up outcome values were handled with the full information likelihood method and the data were analyzed as randomized.

For the primary analysis, ANCOVA models comparing change scores between each group revealed a significant difference in VO_2peak_ improvement between HIIT and MIT, such that MIT improved VO_2peak_ by 11.1% whereas HIIT only improved VO_2peak_ 2.8% on average (P = 0.0185, **Table 3)**. No other significant group differences were observed. In the ITT analysis, diastolic blood pressure became significantly different, while VO_2_ became non-significant. Nominal significance did not change for other variables.

## Discussion

The purpose of this study was to compare the effects of six weeks of HIIT vs MIT for improving cardiometabolic risk factors in a cohort of sedentary, overweight or obese, young men. The primary findings from this study were that young, previously sedentary overweight or obese men randomly assigned to six weeks of MIT had a significantly greater improvement in cardiovascular fitness as compared to the HIIT group, but otherwise displayed no significant difference between groups. Combined, after being randomized to HIIT or MIT, the participants displayed: 1) improved insulin sensitivity, 2) reduced blood lipids, 3) decreased percent body fat, and 4) improved cardiovascular fitness. Overall, these observations suggest that a relatively short duration of either HIIT or MIT training may improve cardiometabolic risk factors in previously sedentary overweight young men, consistent with established cardiometabolic benefits of exercise versus inactivity. However, it is important to note that in the absence of a no-exercise control group we cannot exclude the possibility that these improvements were due to other changes in diet or lifestyle that may be occurring in sedentary young men who have transitioned to a desire to improve their health and fitness.

There have been multiple studies that have shown the ability of HIIT to improve overall cardiovascular fitness [10, 16, 17, 24–26]. Some studies have found a greater improvement in VO_2peak_ in HIIT as compared to MIT [17, 25]; whereas others have found no difference in VO_2peak_ improvement between HIIT and MIT [10, 16, 26]. Similar to these studies we also demonstrate that a short duration of HIIT can lead to significant improvements in VO_2peak_. However, contrary to previous studies [17, 25] we found a greater VO_2peak_ improvement in our MIT group (11.1%) as compared to our HIIT group (2.8%). Improvements in VO_2peak_ have been reported in the ranges of 4–13% after 2–10 weeks of HIIT training in previously sedentary individuals [27]. These differences between studies may be due to differences in exercise intensity and interval duration. Generally, it has been shown that low-volume, all-out-sprint interval training (20 minutes, ~ 250% VO_2peak_) as used in this study yields no significant differences between HIIT and MIT [10, 16, 26], whereas lower intensity intervals performed for longer durations (40–60 minutes, between 80–100% VO_2peak_) consistently yield greater improvements in HIIT compared to MIT [17, 25]. Importantly, while there are varying results between studies, improving overall cardiovascular fitness is critical because low fitness is known to be the strongest predictor for adverse cardiometabolic health outcomes and all-cause mortality [28], and the VO_2peak_ improvements in the MIT and HIIT groups in this study are sufficient to translate into a clinically significant reduction in cardiovascular disease mortality risk [29].

It is critical to recognize early abnormalities of glucose and insulin metabolism in order to prevent progression of type 2 diabetes and cardiovascular comorbidities associated with hyperinsulinemia and glucose intolerance. Exercise training has long been recognized as a means to improve glucose homeostasis via both a short-term, insulin-independent mechanism and a long-term, insulin-dependent mechanism [30, 31]. However, the effect of exercise intensity for improving insulin sensitivity is less understood. For example, two different studies by the same research group have shown that total exercise duration may be more important when assessing insulin sensitivity within 24 hours after the last exercise bout [32], whereas moderate intensity exercise and vigorous intensity exercise may have similar benefits for improving long-term (>24 hrs) insulin sensitivity [33]. The present study assessed insulin sensitivity at least 48-hrs after the last exercise session because we wanted to examine the training effect of exercise, as opposed to the effect of acute muscle contractions, on glucose regulation. We found no significant difference between HIIT and MIT, but did demonstrate an overall 17.6% increase in insulin sensitivity from baseline to post-exercise training in all participants. Thus, it appears that both low-volume HIIT and continuous MIT may provide health benefits that lead to improved glucose tolerance for as long as 48-hrs following the last exercise bout. To date, several studies have shown improvements in glucose homeostasis following HIIT [16–18]. However, because the only two studies to include a MIT exercise group for comparison yielded different results [17, 18], it is difficult to confidently make conclusions on the impact of HIIT compared to MIT on glucose regulation [18]. Future, well-powered randomized controlled trials should be conducted to address these gaps in the literature.

Several recent studies have shown improvements in body composition in both men and women following HIIT [15, 24, 34]. Specifically, a significant increase in fat-free mass and decease in fat-mass has been shown in recreationally active men and women [15, 24], and a significant decrease in whole body and abdominal fat mass was shown in overweight women [34]. However, these results have not been corroborated in all studies as no change in body composition has been observed following HIIT in several other studies [10, 17]. The present study found no differences in changes in body composition between HIIT and MIT, but there was a significant decrease in body fat % from baseline to post-exercise training in all participants. These data suggest that performing relatively little HIIT training as compared to MIT may yield similar improvements in body composition in overweight young men. While variable results have been reported, it cannot be ignored that HIIT requires 20% of the time commitment as MIT and may therefore be a more time-efficient approach for achieving the beneficial effects of exercise on body composition. However, more long term studies comparing HIIT and MIT on body composition measures are warranted before we can confidently prescribe HIIT as an alternative to MIT for improving body composition and long term weight maintenance.

No change in systolic or diastolic blood pressure was observed in the primary analysis of this study. However, the ITT analysis showed a significant decrease in diastolic blood pressure in the MIT compared to the HIIT group. Blood pressure was assessed on average at least 48hrs following the last exercise bout, thus the lack of a decrease was likely due to the timing as exercise has been shown to transiently lower blood pressure for up to 24hrs post-exercise [35]. Indeed, Whyte et al. used a similar protocol as our study and found a significant reduction in blood pressure at 24hrs, but not 72hrs following HIIT [16]. The majority of studies that have demonstrated an improvement in blood pressure following chronic exercise training have assessed blood pressure within 24 hrs of completion of the last exercise bout, thus we chose to assess blood pressure at 48hrs in order to assess more chronic adaptations following exercise because the acute responses have been well documented. We found no significant differences for changes in blood lipid concentrations between HIIT and MIT; however we did find an overall significant decrease in total cholesterol, triglycerides, medium VLDL, and medium HDL from baseline to post-exercise training. There have been several studies examining the effect of HIIT on serum lipids [16, 18, 25, 36]. These studies have generally assessed total cholesterol, HDL-C, LDL-C, and triglycerides. There have been a few reports of an increase in HDL-C following at least 8 weeks of HIIT with no improvements in the MIT groups [18, 36]; however no other changes in blood lipids were observed. We did not find any improvements in HDL-C in this study, but we did see improvements in total cholesterol, triglycerides, medium VLDL particle concentration, and medium HDL particle concentration before and after training in HIIT and MIT combined. More recently it has become evident that lipoprotein subclasses may be more clinically meaningful compared to traditional assessment of serum lipids [37]. For example, different sizes and densities of HDL particles have been differentially associated with CVDs in population studies and CVD mechanisms in ex-vivo studies [38]. Specifically, large HDL particles are associated with a reduction in CVD whereas medium and small HDL particles may be linked to increased risk of CVD. Additionally, small and medium LDL particles appear to be stronger predictors of CVDs compared to larger LDL particles [39]. Given these data, although we did not see a significant change in HDL-C or LDL-C, the decrease in both medium- VLDL and HDL particles and a trend for a decrease in small- HDL particles (P = 0.09) suggests an improved blood lipid profile that favors a reduction in CVD risk.

Strengths of this study included state of the art measures of cardiovascular fitness, body composition, insulin sensitivity, and robust measures of lipoproteins, and supervised exercise training in a clinical setting. A primary limitation in this study was the lack of a no-exercise control group. Thus, we are unable to determine causality in our interpretation of the observed exercise-induced improvements in health parameters. To estimate the effects of MIT and HIIT against a no-exercise control, future randomized controlled studies should be performed for longer durations and include all three groups. However, the purpose of this study was to conduct a head to head comparison of MIT vs HIIT at intensities we hypothesized would result in similar effects, while also having a markedly different time commitment.

In conclusion, the present study demonstrated that HIIT and MIT are both associated with improvements in cardiometabolic health in sedentary, overweight, young men. Similar to many previous investigations, HIIT requires substantially less time commitment compared to continuous MIT, but elicits many of the same improvement in overall health. The only significant difference between groups was a greater improvement in cardiovascular fitness in MIT as compared to HIIT, thus when comparing our HIIT protocol to the MIT protocol used it may be more favorable to utilize MIT when trying to improve overall cardiovascular fitness. While it is premature to conclusively recommend HIIT as an alternative mode of exercise training, the lower time commitment needed to perform HIIT may be more appealing and help to improve exercise adherence. Additionally, the use of HIIT in populations other than athletes or young, healthy individuals is a common concern when considering HIIT as an alternative exercise treatment. However, several recent studies in patients with metabolic syndrome and coronary artery disease have shown that HIIT can be successfully implemented without complications [18, 40, 41]. We also found that HIIT was well tolerated in overweight/obese and sedentary men. Thus, we believe that HIIT may be an ideal mode of training alone or as a compliment to MIT or resistance training. Future research should be conducted to refine training duration and intensity in order to identify optimal doses to target specific health benefits.

## Supporting Information

S1 CONSORT Checklist Consort 2015 Checklist Document. (DOC)

S1 Protocol HIIT MAX Institutional Review Board Approval Document. (DOC)
