# Supplementary material for: High Intensity Interval- vs Moderate Intensity- Training for Improving Cardiometabolic Health in Overweight or Obese Males: A Randomized Controlled Trial
Source: PLoS One. 2015 Oct 21;10(10):e0138853. doi: 10.1371/journal.pone.0138853 (PMC4619258; doi:10.1371/journal.pone.0138853)
Supplement: S1 Protocol — (DOC) [file pone.0138853.s002.doc]

| I | **Human Subjects Protocol (HSP)**  Form Version: June 26, 2012 | 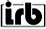 |
| --- | --- | --- |

- **You are applying** for IRB review of the research described in this form.
- **To avoid delay**, respond to all items in order and include all required approvals and documents.
- **To complete the form***,* click the underlined areas and type or paste in your text; double-click checkboxes to check/uncheck. For more tips, see www.uab.edu/irb/forms.
- **Mail or deliver all materials to AB 470**, 701 20th Street South, Birmingham, AL 35294-0104.

**Indicate the type of review you are applying for:**

**x** Convened (Full) IRB *or*

Expedited—See the [Expedited Category Review Sheet,](../expedited-category-review-sheet.doc) and indicate the category(ies) here: 1 2 3 4 5 6 7

**1. IRB Protocol Title: High Intensity Interval vs Moderate Intensity Training: Maximizing the benefits of exercise in overweight adolescents.**

**2. Investigator, Contacts, Supervisors**

**a.** Name of Principal Investigator: **Gordon Fisher**

Degree(s)/Title: **PhD** BlazerID: **grdnfs**

Dept/Div: **Human Studies** Mailing Address: **EB-232J** UAB ZIP: **35294**

Phone: **6-4114** Fax: E-mail: **grdnfs@uab.edu**

**b.** Name of Contact Person: **Amy Thomas** Title: **Study Coordinator**

Phone: **5-9273**

E-mail: **amysusan@uab.edu** Fax:**5-7560**

Mailing Address (if different from that of PI, above): **LHL 448**

**INVESTIGATOR ASSURANCE STATEMENT & SIGNATURE**

By my signature as Principal Investigator, I acknowledge my responsibilities for this Human Subjects Protocol, including:

- Certifying that I and any Co-Investigators or Other Investigators comply with reporting requirements of the UAB Conflict of Interest Review Board;
- Certifying that the information, data, and/or specimens collected for the research will be used, disclosed and maintained in accordance with this protocol and UAB policies;
- Following this protocol without modification unless (a) the IRB has approved changes prior to implementation or (b) it is necessary to eliminate an apparent, immediate hazard to a participant(s);
- Verifying that all key personnel listed in the protocol and persons obtaining informed consent have completed initial IRB training and will complete continuing IRB training each year;
- Verifying that all personnel are licensed/credentialed for the procedures they will be performing, if applicable;
- Certifying that I and all key personnel have read the *UAB Policy/Procedure to Ensure Prompt Reporting of Unanticipated Problems Involving Risks to Subjects or Others to the IRB, Institutional Officials*, *and Regulatory Agencies* and understand the procedures for reporting;
- Applying for continuing review of the protocol at least annually unless directed by the IRB to apply more frequently;
- Conducting the protocol as represented here and in compliance with IRB determinations and all applicable local, state, and federal law and regulations; providing the IRB with all information necessary to review the protocol; refraining from protocol activities until receipt of initial and continuing formal IRB approval.

**Signature of Investigator: Date:**

**c.** List all staff who will be involved with the design, conduct, and reporting of the research, their degree(s) and job title, and any additional qualifications. Include individuals who will be involved in the consent process. *Repeat the table below for each individual.*

*Note.* *For studies involving investigational drugs, include all investigators who will be listed on FDA Form 1572 and attach a copy, if applicable. Send the IRB a copy of Form 1572 anytime you update the form with the FDA.*

| Role: | Co- *-OR-* Other *–AND/OR-* Consent Process |
| --- | --- |
| Full Name: | **David B. Allison** |
| Primary UAB Dept.: (Employer if not UAB) | **School of Public Health Dean’s Office** |
| Degree(s) / Job Title: | **PhD** |
| Additional Qualifications pertinent to the study: | **Advisor** |

| Role: | Co- *-OR-* Other *–AND/OR-* Consent Process |
| --- | --- |
| Full Name: | **Andrew Brown** |
| Primary UAB Dept.: (Employer if not UAB) | **Office of Energetics** |
| Degree(s) / Job Title: | **PhD, Post-doc** |
| Additional Qualifications pertinent to the study: |  |
| Role: | Co- *-OR-* Other *–AND/OR-* Consent Process |
| Full Name: | **Michelle Bohon Brown** |
| Primary UAB Dept.: (Employer if not UAB) | **Nutrition Sci** |
| Degree(s) / Job Title: | **PhD, Post-doc** |
| Additional Qualifications pertinent to the study: |  |
| Role: | Co- *-OR-* Other *–AND/OR-* Consent Process |
| Full Name: | **Karen Keating** |
| Primary UAB Dept.: (Employer if not UAB) | **Office of Energetics** |
| Degree(s) / Job Title: | **PhD, Research Associate** |
| Additional Qualifications pertinent to the study: | **Statistician** |
| Role: | Co- *-OR-* Other *–AND/OR-* Consent Process |
| Full Name: | **Amy Susan Thomas** |
| Primary UAB Dept.: (Employer if not UAB) | **Office of Energetics** |
| Degree(s) / Job Title:  Additional Qualifications pertinent to the study: | **MPH, RD, Study Coordinator**  **Exercise Physiologist** |
| Role: | Co- *-OR-* Other *–AND/OR-* Consent Process |
| Full Name: | **Corey Noles** |
| Primary UAB Dept.: (Employer if not UAB) | **Office of Energetics** |
| Degree(s) / Job Title: | **BS, Research Assistant** |
| Additional Qualifications pertinent to the study: | **Exercise Physiologist** |
| Role: | **Co- -OR-** **Other –AND/OR-** **Consent Process** |
| Full Name: |  |
| Primary UAB Dept.: (Employer if not UAB) |  |
| Degree(s) / Job Title: |  |
| Additional Qualifications pertinent to the study: |  |

**d.** Is the principal investigator a student, fellow, or resident? Yes No

**If Yes**, complete items below and obtain signature of faculty advisor or supervisor:

| Supervisor's Name: |  |
| --- | --- |
| Degree(s) / Job Title: |  |
| Additional Qualifications pertinent to the study: |  |
| Telephone: |  |
| E-Mail: |  |
| **Signature:** |  |

**e.** Describe the principal investigator's activities related to this protocol and provisions made by the PI to devote sufficient time to conduct the protocol:

**As his first funded project as a new Assistant Professor, this project is a priority for the PI. Dr. Fisher will be hands-on for initial testing and exercise training sessions and will personally conduct biochemical analyses in addition to overall implementation of the protocol. He has made adequate allowances in his schedule to conduct this research.**

**f.** Is medical supervision required for this research? Yes No

**If Yes**, who will provide the supervision?

PI will provide *-OR-* Name: Telephone:

If other than PI, obtain signature of person providing medical supervision:

Signature

**g.** Describe the process that ensures that all persons assisting with the research are adequately informed about the protocol and their research-related duties and functions: **All persons assisting with the research will be given a copy of the final study protocol. All persons assisting with the research will receive instruction and training in their particular roles. All persons involved will meet regularly with study investigators during the start-up phase and intervention phase to reinforce information presented in the protocol and present any changes in the protocol that become necessary during conduct of the study.**

**3. Funding**

Is this study funded? Yes No

**If No,** specify that costs of the study will be covered by funds from the UAB department or other source named:

**If Yes**, attach one copy of completed application or request for funding sent to sponsor, and complete a-d.

**a.** Title of Grant or Contract: **High Intensity Interval Versus Moderate Intensity Training: Maximizing the Benefits of Exercise in Overweight Adolescents.**

**b.** PI of Grant or Contract: **Gordon Fisher**

**c.** Office of Sponsored Programs Proposal Number: **NA**
 (*or enter "Pending" and provide upon receipt from OSP*)

**d.** Sponsor, Funding Route (*check and describe all that apply*):

Gov’t Agency or Agencies—Agency name(s):

Department of Defense (DoD): Identify DoD component:

Department of Energy (DOE)

Department of Justice (DOJ)

Department of Education

NIH Coop. Group Trial—Group name:

Private Nonprofit (e.g., Foundation)—Name: **Coca-Cola Foundation**

Industry, investigator-initiated—Name: Describe the funding arrangement:

*Note.* [*Western IRB*](http://www.uab.edu/irb/wirb/wirb-new-review-checklist-uab.doc) *reviews industry-sponsored protocols unless the investigator initiated the research, or the study qualifies for expedited review or involves gene therapy.*

UAB Departmental/Division Funds—Specify:

**4. Conflict of Interest—Human subjects research involving a disclosed financial interest is subject to IRB review following review by the Conflict of Interest Review Board.**

The following definitions are used for Item #4:

***Immediate family*** means spouse or a dependent of the employee. *Dependent* is any person, regardless of his or her legal residence or domicile, who receives 50% or more of his or her support from the public official or public employee or his or her spouse or who resided with the public official or public employee for more than 180 days during the reporting period.

***Financial Interest Related to the Research*** means financial interest in the sponsor, product or service being tested, or competitor of the sponsor.

For each investigator and staff member involved in the design, conduct and reporting of the research (see Items 2.a. and 2.c.) answer the questions below: **(Repeat the section below for each individual)**

***Name:* David B. Allison**

Do you or your immediate family have any of the following? (check all that apply)

|  | An ownership interest, stock options, or other equity interest related to the research of any value. |
| --- | --- |
|  | Compensation related to the research unless it meets two tests:   - Less than $10,000 in the past year when aggregated for the immediate family. - Amount will not be affected by the outcome of the research. |
|  | Proprietary interest related to the research including, but not limited to, a patent, trademark, copyright, or licensing agreement. |
|  | Board of executive relationship related to the research, regardless of compensation. |

***Name:* Gordon Fisher**

Do you or your immediate family have any of the following? (check all that apply)

|  | An ownership interest, stock options, or other equity interest related to the research of any value. |
| --- | --- |
|  | Compensation related to the research unless it meets two tests:   - Less than $10,000 in the past year when aggregated for the immediate family. - Amount will not be affected by the outcome of the research. |
|  | Proprietary interest related to the research including, but not limited to, a patent, trademark, copyright, or licensing agreement. |
|  | Board of executive relationship related to the research, regardless of compensation. |

***Name:* Andrew Brown**

Do you or your immediate family have any of the following? (check all that apply)

|  | An ownership interest, stock options, or other equity interest related to the research of any value. |
| --- | --- |
|  | Compensation related to the research unless it meets two tests:   - Less than $10,000 in the past year when aggregated for the immediate family. - Amount will not be affected by the outcome of the research. |
|  | Proprietary interest related to the research including, but not limited to, a patent, trademark, copyright, or licensing agreement. |
|  | Board of executive relationship related to the research, regardless of compensation. |

**If you checked** **any of the above**, a financial interest disclosure has to be submitted to or currently be on file with the CIRB. A completed CIRB Evaluation has to be available before the IRB will conduct its review.

**5. Locations Involved**

**a.** Describe the facilities available for the conduct of the research. For research on UAB campus, include building names and room numbers: **Exercise Physiology Lab School of Nursing GM019; Core Lab of Nutrition and Obesity Research Center, Webb Building 337; Core Lab of Diabetes Research Center, Webb Building 337; Clinical Research Unit, Jefferson Towers 15.**

**b.** Indicate all "performance sites" that will provide space, services, facilities, potential or actual participants, or other support for this protocol.

The Kirklin Clinic (TKC)

University of Alabama Hospital (UAHosp)

The Children's Hospital of Alabama (TCHA)

Callahan Eye Foundation Hospital (CEFH)

UAB Highlands

Jefferson County Dept. of Health (JCDH)

Birmingham Veterans Affairs Medical Center (BVAMC)

General Clinical Research Center (GCRC)—inpatient

General Clinical Research Center (GCRC)—outpatient

General Clinical Research Center (GCRC) at The Kirklin Clinic (TKC)

Other (i.e., Any performance site not listed above, including those covered by subcontracts related to this protocol)—Describe:**see 5a**

**c.** Is this study a clinical trial requiring clinical services at one of the performance sites listed in Item b above? Yes No

**If Yes**, Fiscal Approval Process (FAP)-designated units complete a FAP submission and send to [fap@uab.edu](mailto:fap@uab.edu). For more on the UAB FAP, see [www.uab.edu/ohr](http://www.uab.edu/ohr).

**d.** Is this a field study? Yes No

**If Yes**, describe the community and include information about how the community will be involved in the design, implementation and analysis of the research. This would include focus groups, training local facilitators/community health advisors:

**e.** Is the study to be undertaken within a school, business, or other institution that does not have an institutional review board? Yes No

**If Yes**, attach a statement of any contacts with and approvals from the appropriate institution officials.

*Note.* *Documentation of all such approvals must be received by the UAB OIRB before IRB approval will be issued.*

**f.** Has this protocol or project been reviewed by another IRB, similar review board, or departmental review committee(s) that authorizes the use of its patient populations? Yes No

**If Yes**, provide name of the review board(s): and for each board listed,

enter either the date of latest approval(s) or “PENDING”: or reasons not approved:. *If this protocol is subsequently rejected or disapproved by another review board, the UAB IRB must be notified promptly.*

***Name:* Michelle Bohon Brown**

Do you or your immediate family have any of the following? (check all that apply)

|  | An ownership interest, stock options, or other equity interest related to the research of any value. |
| --- | --- |
|  | Compensation related to the research unless it meets two tests:   - Less than $10,000 in the past year when aggregated for the immediate family. - Amount will not be affected by the outcome of the research. |
|  | Proprietary interest related to the research including, but not limited to, a patent, trademark, copyright, or licensing agreement. |
|  | Board of executive relationship related to the research, regardless of compensation. |

***Name:* Amy Susan Thomas**

Do you or your immediate family have any of the following? (check all that apply)

|  | An ownership interest, stock options, or other equity interest related to the research of any value. |
| --- | --- |
|  | Compensation related to the research unless it meets two tests:   - Less than $10,000 in the past year when aggregated for the immediate family. - Amount will not be affected by the outcome of the research. |
|  | Proprietary interest related to the research including, but not limited to, a patent, trademark, copyright, or licensing agreement. |
|  | Board of executive relationship related to the research, regardless of compensation. |

***Name:* Karen Keating**

Do you or your immediate family have any of the following? (check all that apply)

|  | An ownership interest, stock options, or other equity interest related to the research of any value. |
| --- | --- |
|  | Compensation related to the research unless it meets two tests:   - Less than $10,000 in the past year when aggregated for the immediate family. - Amount will not be affected by the outcome of the research. |
|  | Proprietary interest related to the research including, but not limited to, a patent, trademark, copyright, or licensing agreement. |
|  | Board of executive relationship related to the research, regardless of compensation. |

***Name:* Corey Noles**

Do you or your immediate family have any of the following? (check all that apply)

|  | An ownership interest, stock options, or other equity interest related to the research of any value. |
| --- | --- |
|  | Compensation related to the research unless it meets two tests:   - Less than $10,000 in the past year when aggregated for the immediate family. - Amount will not be affected by the outcome of the research. |
|  | Proprietary interest related to the research including, but not limited to, a patent, trademark, copyright, or licensing agreement. |
|  | Board of executive relationship related to the research, regardless of compensation. |

*Attach copies of approvals/disapprovals.*

**g.** Will any of the participants be from the Birmingham Veterans Affairs Medical Center? Yes No

**If Yes**, attach VA IRB approval or notification from the VA Research and Development Department that the study has been submitted to the VA IRB for review.

**h.** Will the study be conducted at or recruit participants from the Jefferson County Department of Public Health (JCDH)? Yes No

**If Yes**, attach notification that the protocol has been approved by JCDH or the Alabama Department of Public Health IRB.

**6. Multi-Site Studies**

**a.** Is the investigator the lead investigator of a multi-site study? Yes No

**b.** Is UAB a coordinating site in a multi-site study? Yes No

**c.** If you answered **Yes** to *a* or *b*, describe the management of information obtained in multi-site research that might be relevant to the protection of participants. Include, at a minimum, the following items:

- - IRB approvals from other sites
  - Unanticipated problems involving risks to participants or others. (For example, if there is an unanticipated problem involving risks to participants or others, which site is responsible for reporting it?)
  - Interim results.
  - Protocol modifications.

**7. Drugs:** Will any drugs or supplements be used/studied in this protocol? Yes No

**If Yes**, attach the [Drug Review Sheet](http://www.uab.edu/irb/forms/drug-review-sheet.doc).

**8. Devices:** Will any devices be studied in this protocol or used for a purpose other than for which they were approved by the FDA?  Yes No

**If Yes**, attach the [Device Review Sheet](http://www.uab.edu/irb/forms/device-review-sheet.doc).

**9. Special Approvals**

**a.** Does this project involve the use of radioisotopes? Yes No

**If Yes**, attach documentation of approval from the Radiation Safety Division.

**b.** Does this project include patients with contagious infections (e.g., mumps, measles, chickenpox, TB, meningitis)? Yes No

**If Yes**, attach documentation of approval from Chairman of the Infection Control Committee of the appropriate facilities.

**c.** Does this project involve obtaining remnant biopsy or surgical material from the Department of Pathology or any other source? Yes No

**If Yes**, attach documentation of approval from the entity or individual providing the materials (e.g., the [UAB Division of Anatomic Pathology Release of Pathologic Materials](http://www.uab.edu/irb/forms/release-of-pathologic-materials.doc)).

**d.** Does this project require obtaining any remnant clinical laboratory specimens, body fluids, or microbiological isolates from the Department of Pathology or any other source? Yes No

**If Yes**, attach documentation of approval from the entity or individual providing the materials (e.g., the [UAB Division of Laboratory Medicine Release of Pathologic Materials](http://www.uab.edu/irb/forms/release-of-pathologic-materials2.doc)).

**e.** Does this project use stored (existing) specimens from a repository? Yes No

**If Yes**, attach documentation of approval for use of specimens, and describe how existing specimens are labeled:

**10. Use of Specimens**

Does this project involve collecting specimens from participants and storing them for future research? Yes No

**If Yes**, complete a-h. If no, skip to Item 11

**a.** How will specimens be obtained, processed, distributed, and stored?

**b.**  How will specimens be labeled (e.g., unique identifier, medical record number, Social Security number, name, date of birth)?

**c.**  How will clinical data associated with the specimens be collected and stored?

**d.**  What participant-identifying information will be collected and linked to the specimens?

**e.** What steps will be taken to maximize the confidentiality of linked identifiers? For example, procedures could include using a password-protected computer database to link identifiers, with limited personnel knowledgeable of the password, or coded identifiers released without the ability to link to clinical data (also called "stripped" or "anonymized" specimens).

**f.** Will specimens be shared with other investigators in the future? Yes No

**If Yes**, what identifiers, clinical information and demographic information will be shared; or will the specimens be stripped of identifiers (i.e., anonymized)? Also **if yes**, outline your procedure for assuring IRB approval for release and use prior to release of specimens.

*Note. Investigators who receive and/or use these specimens must document approval from the appropriate IRB(s) before the specimens may be released.*

**g.** Will biological samples be stored for future use? Yes No

**If Yes**, indicate whether they will be used for the disease under study in this protocol or research on other diseases.

**h.** Is genetic testing planned? Yes No

**If Yes**, describe the planned testing here and see "DNA/Genetic Testing" in the Guidebook for consent requirements.

**11. Gene Therapy**

Does this project involve gene therapy or administering recombinant materials to humans? Yes No

**If Yes**, submit the [Gene Therapy Project Review Panel Report](http://www.uab.edu/irb/forms/rprp.doc) –OR- If this is a vaccine trial that is exempt from the NIH Guidelines For Research Involving Recombinant DNA Molecules, submit the [Protocol Oversight Review Form For Clinical Vaccine Trials](http://www.uab.edu/irb/forms/porf-hiv-vaccine.doc).

**12. HIPAA Privacy and Security**

Will the PI or others obtain, review, or make other use of participants' "personal health information" (i.e., information, whether oral or recorded in any form or medium that (a) is created or received by a health care provider and (b) relates to past, present, or future physical or mental health or condition of an individual; or provision of health care; or payment for provision of heath care)? Yes No

**If Yes**, complete a-e as described.

**a.** Will the data/information be stored or managed electronically (on a computer)?

Yes No

**b.** Is the principal investigator requesting that the UAB IRB waive patient HIPAA authorization from another institution or entity (e.g., insurance company, collaborating institution). Yes No

**If Yes**, attach copy of privacy notices from institution/entity, and provide the name of institution/entity:

**c.** Indicate which, if any, of the listed entities below would provide information or maintain health information collected for this protocol and/or where health information that been collected will be stored/maintained.

The Kirklin Clinic

University of Alabama Hospital

The Children’s Hospital of Alabama

Callahan Eye Foundation Hospital

UAB Highlands

Jefferson County Department of Health

School of Dentistry

School of Health Professions

School of Medicine

School of Nursing

School of Optometry

University of Alabama Health Services Foundation

UAB Health Centers

Viva Health

Ophthalmology Services Foundation

Valley Foundation

Medical West - UAB Health System Affiliate

*Health System Information Systems:*

HealthQuest

Cerner Millennium (Lab, Radiology, UED, Surgery)

EMMI - Master Member Index

Horizon - IPV (IVR/CDA/CRIS)

CareFlow Net

Eclipsys (PIN)

IMPACT

None—**If None, skip to Item 13.**

**d.** Indicate which of the listed identifiers would be associated/linked with the protected health information (PHI) used for this protocol.

Names

Geographic subdivisions smaller than a State

Elements of dates (except year) related to an individual

Telephone numbers

Fax numbers

Email addresses

Social security numbers

Medical record numbers

Health plan beneficiary numbers

Account numbers

Certificate/license numbers

Vehicle identifiers and serial numbers

Device identifiers and serial numbers

Biometric identifiers

Web universal resource locators (URLs)

Internet protocol address numbers

Full-face photographic images

Any other unique identifying number—Describe:

*Note.* *Codes are not identifying as long as the researcher cannot link the data to an individual*

None—**If None, skip to Item 13.**

**e.** Choose one plan to describe your use of the personal health information:

The data collected meet the specifications for a “limited data set”

—Attach [Data Use Agreement](http://www.hipaa.uab.edu/pdffiles/DataUseAgreementF232r.pdf) or Business Associate Agreement

Research staff will obtain authorization from each patient to use the information

—Attach [Patient Authorization](http://www.uab.edu/irb/forms/hipaa-authorization-phi-research.doc) form, complete except for patient name and IRB protocol number

PI requests Waiver of Patient Authorization to use the information

—Attach [Waiver of Authorization and Informed Consent](http://www.uab.edu/irb/forms/hipaa-request-for-waiver.doc) form

**PROPOSED RESEARCH**

• The IRB will not accept grant applications and/or sponsor's protocols in lieu of the items as outlined below.

• Do not separate responses from items. Instead, insert your response to each item below the item, keeping the information in the order of this form.

• Number each page of the Human Subjects Protocol (i.e., Page *X* of *Y*).

**13. Purpose—in nontechnical, lay language**

Summarize the purpose and objectives of this protocol, including any related projects, in one short paragraph.

This is a single site, 2 parallel arm, randomized, controlled trial comparing the effectiveness of a High Intensity Interval Training (HIIT) versus a continuous Moderate Intensity Training (MIT) program on cardiovascular and metabolic health outcomes in overweight adolescent males. The study will be conducted over a total of 8 weeks. 36 participants will be enrolled, with 18 randomized to HIIT and 18 randomized to MIT. Multiple measures will be performed at baseline and 6 weeks, including anthropometry, body composition, insulin sensitivity, cardiovascular fitness, free living physical activity, blood pressure, blood assays, and appetite sensations.

**14. Background—in nontechnical, lay language**

Summarize in 2-3 paragraphs past experimental and/or clinical findings leading to the formulation of this study. Include any relevant past or current research by the Principal Investigator. For drug and device studies summarize the previous results (i.e., Phase I/II or III studies).

**Obesity and physical inactivity are associated with decreased insulin sensitivity and the development of type 2 diabetes. It has been reported that the more overweight or obese an individual is, the more likely they are to experience adverse metabolic/cardiovascular outcomes. It is well recognized that weight loss achieved through caloric restriction and/or exercise can improve insulin sensitivity and body composition in obese and overweight individuals; however it has also been shown that approximately one-third of those who lose weight will regain weight within one year following treatment. Therefore, it is important to identify novel strategies that will promote exercise adherence and identify an optimal exercise stimulus to prevent adverse metabolic and cardiovascular outcomes associated with obesity. Low-volume high-intensity interval training (HIIT) (repeated sessions of brief intermittent exercise performed at intensities > 90% VO_2max_) has recently been shown to improve insulin sensitivity, glucose tolerance, cardiovascular fitness, and blood pressure similar to traditional endurance exercise (ET) despite requiring 1 hr of training per week for HIIT vs 6 hrs for ET. Given that ‘lack of time’ remains the most commonly cited barrier to regular exercise participation, HIIT training may be a potent time-efficient strategy to induce similar metabolic and cardiovascular adaptations typically associated with ET. Additionally, while the overall caloric expenditure is significantly less performing HIIT as compared to ET; vigorous exercise has been shown to significantly reduce hunger immediately following the session.**

**15. Participants (Screening and Selection)**

**a.** How many participants are to be enrolled at UAB? **36**

If multi-center study, total number at all centers:

**b.** Describe the characteristics of anticipated or planned participants.

Sex:**Male**

Race/Ethnicity: **Various**

Age:**17-19 yo**

Health status:**Generally good health**

*Note. If data from prior studies indicate differences between the genders or among racial/ethnic groups in the proposed research or if there are no data to support or to negate such differences, Phase 3 clinical trials will be required to include sufficient and appropriate entry of gender and racial/ethnic subgroups so that trends detected in the affected subgroups can be analyzed. If ethnic, racial, and gender estimates are not included in the protocol, a clear rationale must be provided for exclusion of this information. If prior evidence indicates that the results will not show gender or racial differences, researchers are not required to use gender or race/ethnicity as selection criteria for study participants. They are, however, encouraged to include these groups. See Section II. Policy of the NIH POLICY AND GUIDELINES ON THE INCLUSION OF WOMEN AND MINORITIES AS SUBJECTS IN CLINICAL RESEARCH – Amended, October, 2001) for further details.*

**c.** From what population(s) will the participants be derived?
**College and high school campuses in the Birmingham area.**

Describe your ability to obtain access to the proposed population that will allow recruitment of the necessary number of participants:

**We will use flyers, posters, word-of-mouth, and classroom presentations to recruit participants.**

Describe the inclusion/exclusion criteria:

**Inclusion criteria**

- Ages 17-19
- Male
- Body mass index (BMI) (25.0 – 35.0 kg/m^2^)
- Interested in improving health and fitness

**Exclusion criteria:**

- Weight loss or gain of >10% of body weight in the past 6 months for any reason.
- Currently taking medication that suppresses or stimulates appetite.
- History of prior surgical procedure for weight control or liposuction.
- Current smoker.

Any major disease, including:

- - Active cancer or cancer requiring treatment in the past 2 years (except nonmelanoma skin cancer).
  - Active or chronic infections, including self-reported HIV positivity and active tuberculosis.
  - Diagnosed heart conditions.
  - Uncontrolled hypertension: systolic blood pressure 160 mm Hg or diastolic blood pressure 95 mm Hg on treatment.
  - Gastrointestinal disease, including self-reported chronic hepatitis or cirrhosis, any episode of alcoholic hepatitis or alcoholic pancreatitis within past year, inflammatory bowel disease requiring treatment in the past year, recent or significant abdominal surgery (e.g., gastrectomy).
  - Asthma.
  - Diagnosed diabetes (type 1 or 2), fasting impaired glucose tolerance (blood glucose 118 mg/dL), or use of any anti-diabetic medications.
- Conditions or behaviors likely to effect the conduct of the trial: unable or unwilling to give informed consent; unable to communicate with the pertinent clinic staff; unwilling to accept treatment assignment by randomization; current or anticipated participation in another intervention research project that would interfere with the intervention offered in the trial; likely to move away from participating clinics before trial completed; unable to walk 0.25 mile in 10 minutes.
- Currently taking antidepressant, steroid, or thyroid medication, unless dosage is stable (no change for 6 months).
- Any active use of illegal or illicit drugs.
- Excessive alcohol intake defined as an average consumption of 3 or more alcohol containing beverages daily.
- Unwilling to limit alcohol intake to ≤2 drink per day (one drink = 4 oz. wine, 12 oz. beer, or ½ shot of liquor).
- Current exerciser (>30 min organized exercise per week).
- Indication of unsuitability of current health for exercise protocol (PARQ).
- Any other conditions which, in opinion of the investigators, would adversely affect the conduct of the trial.

**d.** If participants will comprise more than one group or stratification, describe each group (e.g., treatment/intervention, placebo, controls, sham treatment) **and** provide the number of participants anticipated in each group.

**18 in the High Intensity Interval Training (HIIT) and 18 in the continuous Moderate Intensity Training (MIT)**

**e.** Indicate which, if any, of the special populations listed below will be involved in the protocol. Include the Special Populations Review Form (SPRF) if indicated.

Pregnant Women: Attach [SPRF—Pregnant Women, Fetuses, Neonates/Nonviable Neonates](http://www.uab.edu/irb/forms/sprf-pregnant.doc)

Fetuses: Attach [SPRF—Pregnant Women, Fetuses, Neonates/Nonviable Neonates](http://www.uab.edu/irb/forms/sprf-pregnant.doc)

Neonates/Nonviable Neonates: [SPRF—Pregnant Women, Fetuses, Neonates/Nonviable Neonates](http://www.uab.edu/irb/forms/sprf-pregnant.doc)

Prisoners: Attach [SPRF—Prisoners](http://www.uab.edu/irb/forms/sprf-prisoners.doc)

Minors (<19 years old): Attach [SPRF—Minors](http://www.uab.edu/irb/forms/sprf-children.doc)

Employees or students at institution where research conducted

Persons who are temporarily decisionally impaired

Persons who are permanently decisionally impaired (e.g., mentally retarded)

Non-English Speakers

**For each box checked,** describe why the group is included **and** the additional protections provided to protect the rights and welfare of these participants who are vulnerable to coercion:**Minors: parental verbal consent will be obtained for interested persons <19 years old prior to screening. In addition, all subjects will be given the clear opportunity to decline participation. Employees/students: the informed consent clearly states the protection of employees and students against career or grade manipulation.**

**f.** List any persons other than those directly involved in the study who will be at risk. If none, enter "None":**None**

**g.** Describe the process (e.g., recruitment, chart review) that will be used to seek potential participants (e.g., individuals, records, specimens). Research recruitment by non-treating physicians/staff may require completion of Partial Waiver of Authorization for Recruitment/Screening. (See http://main.uab.edu/show.asp?durki=61981.)

**Recruitment materials attached.**

**h.** If you will use recruitment materials (e.g., advertisements, flyers, letters) to reach potential participants, attach a copy of each item. If not, identify the source (e.g., databases) from which you will recruit participants.

**We will use flyers, posters, word-of-mouth, and classroom presentations to recruit participants.**

**i.** Describe the procedures for screening potential participants.

**Those that respond to the study telephone number will be asked for parent contact information. Parents will be read the Informed Consent over the phone by study personnel. Pending verbal consent of one parent or guardian (waiver of documentation), subjects will be contacted and screened for initial eligibility via telephone. Eligible subjects will be scheduled for a clinic visit, during which informed assent, fasting blood glucose, assessment of height, weight, and blood pressure, and completion of screening questionnaires (PARQ) will occur. Those subjects that pass the second level of screening will be scheduled for baseline measurements.**

**16. Protocol Procedures, Methods, and Duration of the Study—in nontechnical language**

**a.** Describe the study methodology that will affect the participants—particularly in regard to any inconvenience, danger, or discomfort.

**Participants will have their fingers pricked for blood glucose at the screening visit. Blood will be drawn during baseline and final testing (OGTT) via an antecubital catheter over a 2-hour period. Blood will also be drawn via catheter on Day 1 and Day 42 of the training period. During the VO2 Maximum testing there may be discomfort while wearing the mask to include dry mouth and a feeling of claustrophobia. Exercise training may induce delayed onset muscle soreness and fatigue.**

**b.** What is the probable length of time required for the entire study (i.e., recruitment through data analysis to study closure)?

**1.5 years**

**c.** What is the total amount of time each participant will be involved?

**8 weeks**

**d.** If different phases are involved, what is the duration of each phase in which the participants will be involved? If no phases are involved, enter "not applicable."

**Not applicable**

**e.** List the procedures, the length of time each will take, and the frequency of repetition, and indicate whether each is done solely for research or would already be performed for treatment or diagnostic purposes (routine care) for the population. *Insert additional table rows as needed.*

| Procedure | Length of Time Required of Participants | Frequency of Repetition | Research (Res) –OR- Routine Care |
| --- | --- | --- | --- |
| **Telephone screening** | **15 minutes** | **Once** | Res Routine |
| **Final screening** | **30 minutes** | **Once** | Res Routine |
| **Testing visit 1** | **3.5 hours** | **Twice** | Res Routine |
| **Testing visit 2** | **45 minutes** | **Twice** | Res Routine |
| **Testing visit 3** | **15 minutes** | **Twice** | Res Routine |
| **Clinic visit for HIIT** | **20 minutes** | **18** | Res Routine |
| **Clinic visit for MIT** | **60 minutes** | **30** | Res Routine |

**f.** Will an interview script or questionnaire be used? Yes No

**If Yes**, attach a copy.

**g.** Will participants incur any costs as a result of their participation? Yes No

**If Yes**, describe the reason for and amount of each foreseeable cost.

**h.** Will participants be compensated? Yes No

**If Yes**, complete i-v:

**i.** Type: (e.g., cash, check, gift card, merchandise):**check**

**ii.** Amount or Value: **$600**

**iii.** Method (e.g., mail, at visit): **at visit**

**iv.** Timing of Payments: (e.g., every visit, each month):**$100 after baseline testing; $500 after final testing.**

**v.** Maximum Amount of Payments per Participant:**$600**

**17. Describe the potential benefits of the research.**

**The health benefits of regular exercise are well established. And yet it remains difficult for many persons to find the time and circumstances that lead to engaging in regular exercise of a magnitude and duration generally believed to convey health benefits. Therefore, it is essential to develop novel strategies to help people achieve these goals. Here we propose a time-efficient exercise alternative to reduce the risk of comorbidities associated with adiposity while making adherence easier.**

**18. Risks**

**a.** List the known risks—physical, psychological, social, economic, and/or legal—that participants may encounter as a result of procedures required in this protocol. Do not list risks resulting from standard-of-care procedures. *Note. Risks included in this protocol document should be included in the written consent document.*

**Physical risks include possible adverse reaction to the blood draws. These can include lightheadedness or fainting, along with pain, bruising, or infection at the injection site. During the VO2 Maximum testing there is a rare risk of ischemia or cardiac arrest. During the Wingate Power Test and the exercise training protocol, there is a potential risk of mild muscle soreness, an arrhythmia and fatigue.**

**b.** Estimate the frequency, severity, and reversibility of each risk listed.

**We expect adverse reactions to the blood draws to be rare, not severe, and completely reversible. Any soreness or fatigue related to exercise is not expected to be severe and is also completely reversible.**

**c.** Is this a therapeutic study or intervention? Yes No

**If Yes**, complete the following items:

**i.** Describe the standard of care in the setting where the research will be conducted: **The standard of care for increased fitness in overweight and mildly obese persons is exercise prescription.**

**ii.** Describe any other alternative treatments or interventions: **There are a myriad of exercise modes and programs for persons desiring greater physical fitness and health outcomes.**

**iii.** Describe any withholding of, delay in, or washout period for standard of care or alternative treatment that participants may be currently using: **None. All participants will be exercising.**

**d.** Do you foresee that participants might need additional medical or psychological resources as a result of the research procedures/interventions? Yes No

**If Yes**, describe the provisions that have been made to make these resources available.

**e.** Do the benefits or knowledge to be gained outweigh the risks to participants?

Yes No

**If No,** provide justification for performing the research:

**19. Precautions/Minimization of Risks**

**a.** Describe precautions that will be taken to avoid risks and the means for monitoring to detect risks.

**To prevent risks associated with the blood draws experienced phlebotomists, strict sterile technique, and applying pressure on the injection site will be utilized to minimize pain, infection, and bruising. Participants will be closely monitored during blood draws, and at the first sign of lightheadedness, clamminess, or fainting, they will be placed in a reclined position with legs elevated, and pulse and blood pressure will be assessed until they have recovered fully. To avoid exercise-related risks, all study personnel related to the exercise protocol are exercise physiologists, CPR and defibrillator certified. Subjects will be instructed in technique to avoid injury and minimize soreness. Subjects will be supervised during on-site exercise training.**

**If study involves drugs or devices skip Items 19.b. and 19.c., go to Item 20, and complete the** [**Drug**](http://www.uab.edu/irb/forms/drug-review-sheet.doc) **or** [**Device**](http://www.uab.edu/irb/forms/device-review-sheet.doc) **Review Sheet, as applicable.**

**b.** If hazards to an individual participant occur, describe (i) the criteria that will be used to decide whether that participant should be removed from the study; (ii) the procedure for removing such participants when necessary to protect their rights and welfare; and (iii) any special procedures, precautions, or follow-up that will be used to ensure the safety of other currently enrolled participants.

**(i) Any participant experiencing a documented injury or other physical condition, the participant and the study coordinator can assess the situation and determine if the participant wishes to continue. If symptoms do not improve, the participant will be removed from the intervention. (ii) Participants requiring removal from the study will be notified promptly and instructed to discontinue the intervention (exercise). Referral to their primary care provider will be made if necessary, and the participant will be provided with results of any tests or measurements that are available as a result of their participation in the study. (iii) All currently enrolled participants will be continually monitored for adverse reactions or other problems.**

**c.** If hazards occur that might make the risks of participation outweigh the benefits for all participants, describe (i) the criteria that will be used to stop or end the entire study and (ii) any special procedures, precautions, or follow-up that will be used to ensure the safety of currently enrolled participants.

**(i) If a significant percentage of participants (at least 10%) of participants experience a serious adverse event, the study would be stopped. (ii) If the study were to be stopped, all currently enrolled participants would be contacted by telephone promptly and assessed for adverse reactions, as well as be scheduled for a final clinic visit for further assessment.**

**20. Informed Consent**

**a.** Do you plan to obtain informed consent for this protocol? **Yes** **No**

**If Yes,** complete the items below.

**If No,** complete and include the [Waiver of Informed Consent](http://www.uab.edu/irb/forms/request-consent-waiver.doc) or [Waiver of Authorization and Informed Consent](http://www.uab.edu/irb/forms/hipaa-request-for-waiver.doc), as applicable.

**b.** Do you plan to document informed consent for this protocol? **Yes** **No**

**If Yes,** complete the items below.

**If No,** complete the items below **and** include the [Waiver of Informed Consent Documentation](http://www.uab.edu/irb/forms/request-consent-waiver-document.doc).

**c.** How will consent be obtained? **The study coordinator will present the study on the phone to the parent of the potential participant <19 years old. Informed consent (waiver of documentation) will be obtained at that time. Next, the study coordinator will present the study at the screening visit and ample time will be allotted for all potential participants to ask questions and have them answered by study personnel. The consent form will be reviewed page by page, and again time will be allotted for questions and answers. Finally, when the study protocol and consent have been reviewed and all questions have been addressed, individuals agreeing to participate will sign the consent form (those <19yo will be assenting). Each participant will be emailed a copy of their signed consent form.**

**d.** Who will conduct the consent interview? **The study coordinator**

**e.** Who are the persons who will provide consent or permission? **Participants or their parent**

**f.** What steps will be taken to minimize the possibility of coercion or undue influence? **The study coordinator will stress that participation is strictly voluntary and is in no way required in order to receive any type of services at UAB. Only the study coordinator, witness, and potential participant will be involved in the consent process. No other parties will discuss the research with the participant or attempt to exert any undue influence.**

**g.** What language will the prospective participant or the legally authorized representative understand? **English**

**h.** What language will be used to obtain consent? **English**

**i.** If any potential participants will be, or will have been, in a stressful, painful, or drugged condition before or during the consent process, describe the precautions proposed to overcome the effect of the condition on the consent process. If not, enter "no such effect."

**No such effect.**

**j.** If any project-specific instruments will be used in the consenting process, such as flip charts or videos, describe the instrument(s) here, and provide a copy of each. If not, enter "not used."

**Not used.**

**k.** How long will participants have between the time they are told about the study and the time they must decide whether to enroll? If not 24 hours or more, describe the proposed time interval and why the 24-hour minimum is neither feasible nor practical. **Participants will be told about the study in their telephone screening interview; again at the screening visit. The screening consent and visit will take place at least 24 hours after the phone explanation. The study consent and baseline visit will take place at least 24 hours after the screening visit. Additionally, the study consent form will be emailed on the same day as the screening telephone visit, to allow additional time for the participant to review before signing. Parents of interested participants , 19yo will be told about the study on the phone, and allowed whatever length of time they desire to decide. Parents will also be emailed a copy of the informed consent.**

**21. Procedures to Protect Privacy**

Describe the provisions included in the research to protect the privacy interests of participants (e.g., others will not overhear your conversation with potential participants, individuals will not be publicly identified or embarrassed).

**Telephone screening will be conducted in the study coordinator’s private office. Webb Building has several small private rooms for participant interviews. No sensitive conversation will take place in public areas where the discussion may be overheard. Clinic staff will not make any statement where others can hear that might reveal personal information about the participant, study requirements, etc,**

**22. Procedures to Maintain Confidentiality**

**a.** Describe the manner and method for storing research data and maintaining confidentiality. If data will be stored electronically anywhere other than a server maintained centrally by UAB, identify the departmental and all computer systems used to store protocol-related data, and describe how access to that data will be limited to those with a need to know.

**Paper records will be stored in a locking file cabinet in a locked office in the Exercise Physiology Lab, which is secured at all times (access restricted by key card). Electronic data will be stored on the School of Public Health server and computers, which are firewall protected, encrypted, and password-restricted. The servers are monitored at all times for outages. Secured login IDs, granted on a need-to-know basis, are required to access confidential information.**

**b.** Will any information derived from this study be given to any person, including the subject, or any group, including coordinating centers and sponsors? Yes No

**If Yes**, complete i-iii.

**i.** To whom will the information be given?

**ii.** What is the nature of the information?

**iii.** How will the information be identified, coded, etc.?

**23. Additional Information**

In the space below, provide any additional information that you believe may help the IRB review the proposed research, or enter "None."

**None**
